# Health economic evaluations of myasthenia gravis: a systematic review

**DOI:** 10.3325/cmj.2025.66.436

**Published:** 2025-12

**Authors:** Frenki Çipi, Chantal Koloneci, Artejana Marku, Kamila Domi, Xhoana Tahiraj, Visar Malaj, Malvina Hoxha

**Affiliations:** 1 Faculty of Pharmacy, Catholic University Our Lady of Good Counsel, Tirana, Albania; 2 Department of Economics, University of Tirana, Tirana, Albania; 3 Department of Chemical-Toxicological and Pharmacologic Evaluation of Drugs, Faculty of Pharmacy, Catholic University Our Lady of Good Counsel, Tirana, Albania

## Abstract

**Aim:**

To systematically review the latest pharmacoeconomic evidence regarding the costs associated with myasthenia gravis (MG) care and treatment.

**Methods:**

We searched the National Health Service Economic Evaluation Database and PubMed for articles in English from any country reporting health economic analyses of pharmacological treatments, hospitalization, or surgical procedures related to MG.

**Results:**

The study included 31 articles showing a considerable variability in the costs and cost-effectiveness of MG treatments across regions, therapies, and health care settings. Traditional therapies such as plasma exchange (PLEX) and intravenous immunoglobulin (IVIg) differ notably in cost, with PLEX generally being less expensive. Newer clinically effective treatments such as efgartigimod and eculizumab raise concerns about their economic sustainability, whereas rituximab might be a more affordable alternative in certain contexts.

**Conclusion:**

Future research should compare cost-effectiveness across health care systems, incorporating local pricing and reimbursement, and collecting real-world data.

Myasthenia gravis (MG) is an autoimmune disorder affecting synaptic transmission at the neuromuscular junction (1). It is characterized by autoantibodies that target postsynaptic proteins such as acetylcholine receptor (AChR), muscle-specific kinase (MuSK), lipoprotein-related protein 4 (LRP4), or agrin. This autoimmune response disrupts neuromuscular signaling, resulting in muscle weakness and fatigue. The disease is heterogeneous and classified based on antibody specificity, thymic pathology, genetic factors, treatment response, and clinical presentation (2-4).

MG initially manifests in the extraocular musculature and remains confined to ocular involvement in approximately 15% of patients. In the rest, the disease progresses to a generalized form, typically spreading in a descending pattern to affect the bulbar muscles, followed by the neck, proximal limbs, and, in some cases, the respiratory muscles. Severe bulbar or respiratory involvement may lead to myasthenic crisis, a critical condition that often necessitates endotracheal intubation and mechanical ventilatory support (5). MG affects 150 to 250 individuals per million people, with an annual incidence of 8 to 10 cases per million people (6).

Individuals with MG are divided into subgroups according to serum antibodies and clinical features. These subgroups comprise early and late-onset, MuSK-positive, antibody-negative, and LRP4-positive (3,7). Early-onset MG, presenting before 50 years of age, is characterized by AChR antibodies, female predominance, thymic hyperplasia, and responsiveness to thymectomy. Late-onset MG, occurring after 50 years of age, shows male predominance, minimal thymic changes, and limited thymectomy benefit. Thymoma-associated MG is a paraneoplastic form with generalized symptoms and near-universal AChR antibody presence. MuSK-associated MG predominantly affects bulbar muscles, lacks thymic abnormalities, and responds poorly to thymectomy. LRP4-associated MG mainly affects women with mild ocular or generalized symptoms and variable thymic pathology. Antibody-negative MG lacks detectable AChR, MuSK, or LRP4 antibodies but may harbor low-affinity or unidentified autoantibodies. Ocular MG is restricted to eye muscles, often with AChR antibodies, and rarely generalizes after two years (3,8). Treatment approaches are typically divided into two categories: symptomatic therapies, which aim to enhance neuromuscular transmission, and immunosuppressive therapies, which target the underlying immune-mediated pathology of MG (4).

The primary symptomatic treatment, effective across all disease subgroups, is that with acetylcholinesterase inhibitors, especially pyridostigmine bromide (4,5). Recent therapeutic advancements in MG have extended beyond conventional immunosuppressive agents and symptomatic management. Complement inhibitors, including eculizumab, zilucoplan, and ravulizumab have demonstrated significant efficacy in patients with anti-AChR antibody-positive generalized MG. B-cell depletion therapies such as rituximab have shown particular benefit in muscle-specific kinase antibody-positive cases. Fc receptor (FcRn) antagonists, such as efgartigimod and rozanolixizumab, effectively reduce circulating pathogenic IgG antibodies. Innovative strategies, including chimeric antigen receptor T cell therapy and autologous hematopoietic stem cell transplantation, aim to restore immune tolerance in refractory cases. Therapeutic plasma exchange (PLEX) and intravenous immunoglobulin (IVIg) remain indispensable in the acute management of MG. Corticosteroids and immunosuppressive drugs continue to represent foundational treatments. Thymectomy remains a recommended intervention in selected patient populations, particularly those with early-onset, AChR-positive MG. Finally, emerging combination regimens integrating biological and immunosuppressive therapies are being explored to optimize clinical outcomes while minimizing long-term adverse effects (8). Existing studies on the economic burden of MG are limited by small samples, single-country data, and incomplete cost analyses. This study attempts to fill these gaps by providing an up-to-date systematic review with large-scale health economic data on MG including those from different countries and health care settings and those related to different treatments. The aim of this systematic review is to present current pharmacoeconomic evidence on MG health care and treatment costs.

## Methods

### Literature search

This systematic review followed the Preferred Reporting Items for Systematic Reviews and Meta-Analyses (PRISMA) guidelines (9,10). We searched PubMed between inception and March 2025, applying no date restrictions. The following keywords were used: (“myasthenia gravis”) and (“economic evaluation,” OR “cost,” OR “cost benefit,” OR “cost-effectiveness analysis,” OR “cost analysis,” OR “health economic,” OR “cost utility,” OR “direct cost,” OR “indirect cost”).

### Research methodology

We included articles in English from any country that reported any type of health economic analyses regarding pharmacological treatments, hospitalization, or surgical procedures related to MG. Abstracts, reviews, systematic reviews, conference papers, protocols, and editorials were excluded. All articles were screened independently by two reviewers (F.Ç, C.K), and consensus was achieved through discussion.

Original studies on MG were considered suitable if they included a complete or partial economic assessment comparing interventions and comparators based on outcomes and expenses. All articles focusing on MG as the primary outcome but not including interventions related to treatment, hospitalization, or surgery, or not reporting health economic data were excluded.

Study participants consisted of individuals diagnosed with MG. Research involving theoretical cohorts was also considered. The interventions were broadly defined to encompass all treatment strategies, such as pharmacological therapies and surgical procedures. Surgical interventions (robotic-assisted thoracic surgery, median sternotomy, total thymectomy [TT], open thymectomy, minimally invasive surgery, video-assisted thoracoscopic surgery) and pharmacological therapies (acetylcholinesterase inhibitors, PLEX, IVIg/subcutaneous immunoglobulin [SCIg], prednisone, azathioprine, mycophenolate mofetil, cyclosporine, pyridostigmine, methotrexate, rituximab, eculizumab, and efgartigimod) served as interventions/comparators.

### Data acquisition

We gathered data on the country, year of publication, study type, target group, population, discount rate, intervention, comparators, intervention duration, perspective, time range, outcomes, incremental cost-effectiveness ratios (ICERs), quality-adjusted life years (QALYs), and/or disability-adjusted life years (DALYs), and current cost values in the original and current currency. The original cost values were first adjusted for inflation to reflect 2025 prices using the relevant national inflation indices from the year when each study was conducted. Three authors (C.K, A.M, X.H.T) extracted the information.

### Data synthesis

Studies were categorized into cost-effectiveness analysis (CEA) and non-CEA groups. General characteristics and economic data of the non-CEA group are shown in Supplemental Table 1(Supplementary Table 1) and Supplemental Table 2(Supplementary Table 2), respectively, while general characteristics and economic data of the CEA group are shown in Table 1 and Table 2, respectively. Differences were independently reviewed and resolved through discussion between F.Ç and M.H.

**Table 1:** General characteristics of all the included cost-effectiveness studies*

No	Reference	Country	Type of pharmacoeconomic study	Population	Intervention	Comparator/s	Discount rate	Duration	Perspective
1	Lien P. et al, 2024 (22)	USA	Cost-effectiveness (CEA)	Hypothetical cohort of patients with anti-acetylcholine receptor antibody-positive generalized myasthenia gravis (gMG)	Eculizumab; efgartigimod – both added to conventional therapy	Conventional therapy alone	3%	Lifetime horizon modeled with 4-weeks cycle lengths.	Healthcare system
2	Tice J.A. et al, 2022 (23)	USA	CEA	20 000 patients with gMG	Eculizumab (REGAIN) and efgartigimod (ADAPT) treatment	Conventional therapy alone	N.R.	4 weeks of therapy followed by a minimum 4-week break (2 y)	Healthcare system
3	Chicaiza-BecerraL. A. et al, 2012 (41)	Colombia	CEA	Patients with MG without thymoma	Thoracoscopic thymectomy	Medical treatment alone	3%	Lifetime horizon of 54 y	Healthcare system
4	Engebretsen I. et al, 2023 (31)	Norway	CEA	5.5 million Norwegian citizens	Eculizumab + conventional therapy vs conventional therapy alone	Conventional therapy (IVIG), (PLEX)	N.R.	2008–2021	Healthcare system

*Abbreviations: CEA – cost-effectiveness analysis; gMG – generalized myasthenia gravis; IVIg – intravenous immunoglobulin; N.R.– not reported; PLEX – plasma exchange.

**Table 2:** Economic data from all the included cost-effectiveness studies*

No	Reference	Country	Type of pharmacoeconomic study	ICER/QALY/QALD/LLY	Outcomes	Current values in original currency	Current values in current (EUR) currency
1	Lien P. et al, 2024 (22)	USA	Cost-effectiveness (CEA)	Direct costs: eculizumab ICER: $3 338 000/QALY efgartigimod ICER: $1 987 000/QALY Indirect costs: eculizumab ICER: $3 310 000/QALY efgartigimod ICER: $1 959 000/QALY	Efgartigimod shows greater health benefit, with higher incremental QALY gain (3.24 vs 1.56 for eculizumab). Efgartigimod is more cost-effective, having a lower ICER than eculizumab.	Efgartigimod and eculizumab are above the conventional cost-effectiveness threshold of $206 600/QALY (adjusted from $200 000).	Efgartigimod and eculizumab exceed the typical cost-effectiveness threshold of approximately €190 000 per QALY
2	Tice J.A. et al, 2022 (23)	USA	CEA	N.R.	QALY gain: efgartigimod 1.27 vs eculizumab 1.13 Cost-effectiveness Cost per QALY: efgartigimod: $2.08M/QALY eculizumab: $5.2M/QALY Drug cost: eculizumab: $855 400 efgartigimod: $692 700	Cost of treatment: eculizumab: $941 940 efgartigimod: $762 000	Treatment costs: eculizumab: €799 000 efgartigimod: €646 500
3	Chicaiza-Becerra, L. A. et al, 2012 (41)	Colombia	CEA	ICER = Col $1 129 531/LYG without 3% discounting, with discounting ICER = Col $805 179/LYG	Thoracoscopic thymectomy improves survival compared with medical therapy alone Long-term costs are lower with surgery	ICER = Col $1 457 094/LYG without 3% discounting; with discounting, ICER = Col $1 037 682/LYG.	ICER (no 3% discounting): ≈ €350 000 per LYG ICER (with 3% discounting): ≈ €249 000 per LYG
4	Engebretsen I. et al, 2023 (31)	Norway	CEA	Lost life years and reduced quality of life account for 50.5%	Annual societal cost per patient was EUR 24 743.	Annual societal cost per patient: €26 722 Direct medical costs: €3874 (14.5%) Productivity loss: €9353 (35%)	Annual societal cost per patient: €26 722 Direct costs: €3874 (14.5%) Productivity loss: €9353 (35%)

*Abbreviations: CEA – cost-effectiveness analysis; ICER – incremental cost-effectiveness ratio; LYG – life years gained; LLY – life years lost; N.R. – not reported; QALD – quality-adjusted life day; QALY – quality-adjusted life years.

### Outcomes

The primary outcomes in the CEA studies were the incremental QALYs gained and the ICERs of efgartigimod vs eculizumab. Non-CEA studies focused on direct and indirect medical costs associated with rituximab, PLEX, intravenous immunoglobulin, and hospitalization, with an emphasis on cost variations due to disease severity and regional differences.

## Results

### Summary of the included studies

**Figure 1:**
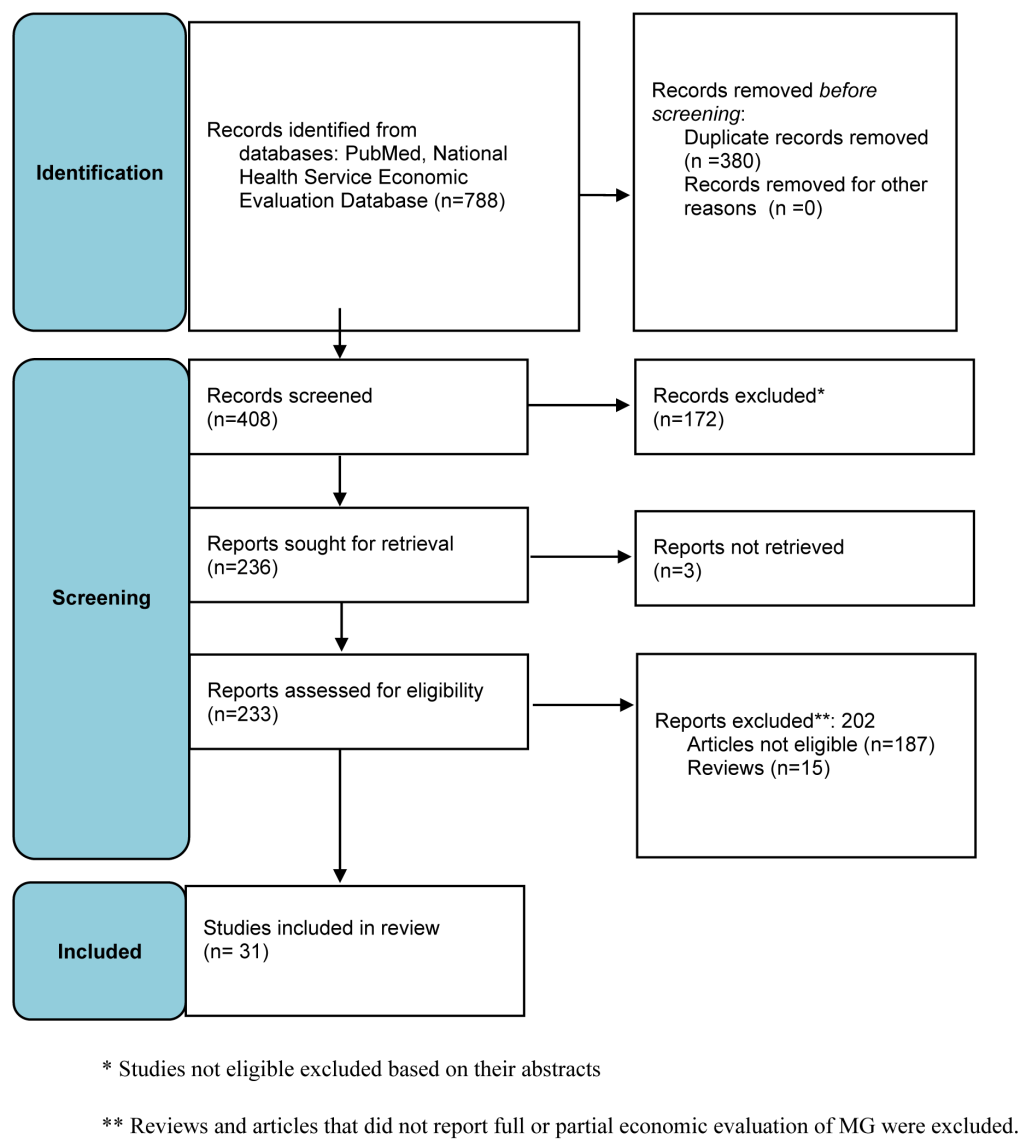
PRISMA flow diagram of literature search and study selection process.

Out of the identified 788 articles, 380 duplicates were removed (Figure 1). Overall, 172 articles were excluded after screening the titles and abstracts, and 202 more were excluded as they did not meet the eligibility criteria. Only 31 studies were included in the final sample.

Geographically, 13 studies were conducted in the USA (11-23), 4 in China (24-27), 2 in Germany (28,29), 2 in Norway (30,31), and 2 across Denmark, Finland, and Sweden (32,33). One study originated from each of the following countries: Portugal (34), Bulgaria (35), the Czech Republic (36), Italy (37), Canada (38), India (39), England (40), and Colombia (41). The publication years ranged from 2009 to 2025. The currencies used for evaluation were US dollars (US$), euros (€), Indian rupees (INR), New Taiwan dollars (NT$), Canadian dollars (CAN$), and British pounds (£).

Among the 31 eligible papers, 11 were cost analysis studies, 8 studies performed cost-description analysis, 5 performed cost-of-illness analysis, 4 performed cost-effectiveness analysis, 2 performed cost-minimization analysis, and one study performed a cost-utility approach. A discount rate of 3% was reported by two studies (22,32). ICER was reported in two studies (22,41).

### Conventional therapies

A cost-minimization study by Heatwole et al showed IVIg to be less expensive ($78 814) than PLEX ($101 140) (11). When converted to the current (2025) euro, the average short-term cost per patient was approximately €121 400 for PLEX and €94 600 for IVIg, with the latter offering average savings of around €26 800 per patient. In the study by Enkhuizen et al, IVIg had an even lower mean annual cost/patient (£1514 or €1786 [2025 euro]) than PLEX (£4233 or €4994 [2025 euro]) (40). In the Czech Republic, both therapies had high annual costs, but IVIg surpassed PLEX (€20 700 and €18,206, respectively) (36).

In Canada, PLEX was the most cost-minimizing option ($6271 vs $8309 for IVIg) (38). According to Guptill et al, PLEX was also cheaper ($949 for a single session) than IVIg ($4663) (17). Ting et al reported that MG-related health care costs among all 1498 patients who did not experience an MG exacerbation were $69 206 ($12 201) per patient over the two-year follow-up period (42). Among patients receiving chronic IVIg only, total MG-related health care costs per patient were $243 849 ($170 928), with mean (median) inpatient, outpatient, and pharmacy costs of $20 611 ($20,61), $170 928 ($121 530), and $52 311 ($547), respectively (42).

In two studies performed by Zhdanava et al, IVIg contributed to 85% of the annual pharmacy and medical costs ($19 573 and $17 949, respectively) (18,21). Additionally, IVIg patients were classified as high-cost patients with annual expenses exceeding $110 000. In Norway, a small group of patients treated with IVIg also reported high costs (€95,364), compared with patients not receiving IVIg (€28,952) (30). According to Sonkaret al, both PLEX and IVIg significantly increased the median annual cost from INR 61 390.5 (US$ 911.6) to INR 378 930 (US$ 5627) (39).

Thymectomy was also a cost driver, with direct costs ranging from INR 37 000 to 200 000 (median INR 45 000, US$ 668). In patients with thymectomy history, Ignatova et al reported higher mean costs at €3047 compared with €825 in patients with no thymectomy history (35). Castillo et al found varying total costs for different thymectomy procedures in the USA: $18 431 (or €19,200, adjusted to 2025 currency) for minimally invasive thymectomy and $22 121 (or €23 000 adjusted to 2025 currency) for open thymectomy (13). Chicaiza-Becerra et al used a different approach for thoracoscopic thymectomy, a cost-of-illness (CoL) study type (41). TT was the most cost-effective alternative compared with medical treatments and open thymectomy, with a CoL $1 129 531/ life years gained (LYG) (CoL $805 179/LYG with a 3% discount rate) compared with an ICER of CoL $1 920 620/LYG.

### Biological therapies

Three studies compared the cost-effectiveness of eculizumab and efgartigimod to conventional therapies. Tice et al found efgartigimod to have a greater QALY gain (+1.27) and a lower ICER ($2.08M/QALY) than eculizumab (+1.13 QALY; $5.2M/QALY) (23). However, both treatments surpassed the cost-effectiveness limit of US standards of $100K-$150K per QALY. According to Tice et al, efgartigimod led to greater improvements in MG activities of daily living (MG-ADL) and MG quality of life 15-item scale (MG-QOL-15r) scores, while eculizumab had high annual/patient costs of €24 743 (42).

In a comparative lifetime analysis by Lien et al, eculizumab was less expensive than efgartigimod, but still significantly pricier than conventional therapies, $5.5 million (11.85 QALYs) vs $308 000 (10.29 QALY with an ICER $3.34 million/QALY), and $6.77 million (13.22 QALY) vs $322 000 (9.98 QALY with an ICER $1.99M/QALY), respectively (22) When converted to 2025 euros, both treatments exceeded the typical cost-effectiveness threshold of approximately €190 000 per QALY.

In a cost-utility study by Peres et al, the annual cost/patient of rituximab decreased over a five-year period from €17 967 to €8881 (34). The outcome of the treatments was a QALY gain of +1.76, along with improvements in MG-QOL-15 (−20 points) and EQ-5D (+0.492).

Rituximab costs were considerably lower in a larger cohort analyzed by Enkhuizen et al (£1811/patient/year) (40). Yet, the costs increased significantly when combined with other therapies like IVIg, PLEX, or mechanical ventilation, a finding that was also observed by Philips et al (12). Lehnerer et al reported that rituximab or eculizumab increased the inpatient costs to €38,669/year compared with €2980 for standard treatments (28).

*Economic burden*

The highest hospitalization costs were reported in the USA, with expenses increasing from $48 024 in 2003 to $98 795 in 2013 (20). According to Guptill et al, MG patients had a higher annual health care cost ($20 190) than the control group ($4515) (19). Pharmacy expenses were a major cost driver ($9012/y), followed by hospitalization and outpatient care. In Taiwan, the mean total annual cost for MG patients was NT$135 219 (US$4456) compared with NT$40 904 (US$1348) for non-MG controls, with inpatient costs comprising the largest portion of expenses (25).

In Germany, the annual cost was €14,940, of which €11 840 accounted for direct costs and pharmaceutical treatments at €1800/y (29). In Italy, the mean annual cost was €3771/patient, also driven by hospitalization (€2198) and medication (€1148) costs (37). In a study by Piehlet al, Denmark had the highest health care cost (€12,185), followed by Finland (€9036) and Sweden (€5997). The majority of the costs amounted to inpatient care (32). In contrast, China reported the lowest direct annual costs at $1860.2, with median hospitalization costs of $1037 (24,27).

In a cohort of 41 940 gMG patients, it was found that newly diagnosed (ND) patients had slightly higher mean all-cause costs ($26 419) than previously diagnosed (PD) patients ($24 941), with the highest costs reported for exacerbation and post-crisis cases (12). The use of IVIg and SCIg led to direct costs ranging from $9186 to $21 550. Similarly, in a smaller cohort by Cai et al, ND patients had almost double first-year all-cause costs of preexisting MG, €14 796 (€9819 medical expenses) vs €7374 (€5931 medical expenses) (33). Costs decreased by the second year and became more comparable between groups.

The economic burden of MG was also influenced by mechanical ventilation. Admissions for ventilated patients rose by 53% from 1991-1992 to 2001-2002 (15). Hospitalization cost/patient increased from $84 100 to $118 000, and daily charges rose from $3932 to $5500. Mechanical ventilation was likewise recognized as an important cost driver (39).

Medical expenses were additionally influenced by insurance types. According to Pisc et al, commercial patients with at least one exacerbation event had higher all-cause costs ($41 194 vs $34 802) and MG-related costs ($39 079 vs $33 446) than Medicare patients, especially in the emergency department, intensive care unit, and outpatient care (14). Medicare inpatient costs increased overtime, but they decreased for commercial patients. In line with a study from the south of China, patients with job-based insurance had lower outpatient ($170.98) and inpatient ($3539.27) costs than patients with residence-based insurance ($209.34 and $4224.08, respectively) (26).

## Discussion

This systematic review compiles available health economics data, including both full and partial economic evaluations of MG interventions across various global contexts. It covers different aspects such as MG pharmacological treatment, hospitalization costs, and surgical interventions. Due to the heterogeneity of study designs, cost perspectives, and outcome measures, a meta-analysis was not feasible.

Our systematic review highlights a considerable variability in the cost and cost-effectiveness of MG management strategies across different regions, therapies, and health care settings. Conventional therapies such as IVIg and PLEX remain widely used but show inconsistent cost profiles depending on country-specific health care systems and pricing structures. While in most settings PLEX was generally less expensive than IVIg (11,12,17,39), this trend was reversed in some studies from England and specific US hospitals (11,38). These inconsistencies may reflect differences in local reimbursement systems, hospital billing practices, and frequency or duration of treatment courses (36,38).

Despite their high cost, conventional therapies continue to be standard care for exacerbations, although studies showed significant cost spikes associated with chronic or recurrent use, particularly with IVIg (4,5,16,25). The presence of high-cost outliers, like chronic IVIg use or patients requiring ICU admission, underscores the economic strain such therapies may pose, especially in low-to-middle-income countries (2,9). Furthermore, surgical interventions like thymectomy demonstrated variable direct costs depending on the technique (minimally invasive, thoracoscopic, or open) (35), though thoracoscopic thymectomy offered a more cost-effective profile in one cost-of-illness analysis (41).

In contrast, the emergence of biological therapies such as eculizumab, efgartigimod, and rituximab brings new considerations to MG pharmacoeconomics. While efgartigimod showed better cost-effectiveness metrics than eculizumab (23), both treatments greatly exceeded standard willingness-to-pay thresholds in the US and EU, which raised concerns about their economic sustainability despite clinical benefits (22,23). Rituximab, however, stood out as a relatively cost-saving biological (34,40). Nonetheless, the economic advantage of rituximab diminished when used in combination with other intensive therapies, which emphasizes the need for clear treatment algorithms that consider both efficacy and economic burden (12,28,34,40).

The burden of MG on national health care systems remains substantial, with hospitalization, inpatient care, and pharmacologic treatments being consistent cost drivers across nearly all studies. Notably, countries with higher health expenditures, such as the US (11-23) and Scandinavian countries (32,33), reported higher per-patient costs than China and Italy (24-27,37). This discrepancy may be attributed to differences in health care funding models, access to biologics, and national pricing policies. Moreover, insurance type and policy structures, such as Medicare, commercial insurance, or job-based plans, influenced MG-related expenses, as reflected in US and Chinese cohorts (14,17,25,26).

Newly diagnosed MG patients, especially those presenting with crises or requiring mechanical ventilation, incurred higher first-year treatment costs (15). This aligns with the understanding that disease severity at onset often necessitates aggressive and costly interventions, the costs of which taper as the disease clinically stabilizes. Mechanical ventilation in particular significantly increased hospitalization expenses and remains a notable cost driver in MG management (39).

Taken together, the data reinforce the complexity of economic evaluation in MG treatment. The diversity of cost types (direct vs indirect), perspectives (payer vs societal), and outcomes (QALY, ICER, LYG) make cross-study comparison challenging (32). Yet, our identification of consistent patterns, such as the high cost of biologics, the variability in conventional therapy costs, and the disproportionate economic burden on severely affected or newly diagnosed patients, can help guide policymakers and clinicians in designing cost-conscious pathways.

Several limitations of this review should be acknowledged. The heterogeneity in study designs, cost perspectives, outcomes, and geographic settings limits the study’s comparability and generalizability. Variations in health care system structures and reimbursement policies significantly influenced the reported costs, making direct cross-country comparisons challenging. Additionally, our search was limited to articles published in English and conducted exclusively through PubMed. Another limitation is the scarcity of real-world, long-term cost-effectiveness data for newly introduced therapies. Finally, there was a notable underrepresentation of studies from low- and middle-income countries, such as India, China, Bulgaria, and Colombia, which highlights a gap in globally representative data (24-27,35,39,41).

In conclusion, this review provides updated insights into the economic aspects of MG treatment. The included studies revealed considerable variability in treatment costs across countries and therapies. Traditional approaches such as IVIg and PLEX exhibited notable cost differences, with PLEX often being more affordable. Newer treatments, efgartigimod and eculizumab, while showing clinical efficacy, raise concerns about their economic sustainability. Rituximab emerged as a potentially more economic option in specific settings. However, the limited number of high-quality pharmacoeconomic studies remains a barrier to informed health care decision-making. Future research should compare cost-effectiveness across health care systems, consider local pricing and reimbursement, and gather more real-world data, especially in low- and middle-income countries, to support equitable and informed decision-making. These evidence gaps must be addressed to develop sustainable and accessible treatment strategies for MG.
