# Supplementary material for: Health economic evaluations of myasthenia gravis: a systematic review
Source: Croat Med J. 2025 Dec;66(6):436–45. doi: 10.3325/cmj.2025.66.436 (PMC12836010; doi:10.3325/cmj.2025.66.436)
Supplement: Supplementary Table 1 [file CroatMedJ_66_s003.pdf]

Supplemental Table 1. General characteristics of included health economic studies (excluding cost-effectiveness analyses)

No.	Reference	Country	Type of pharmacoeconomic study	Perspective	Population	Discount Rate	Intervention	Comparators	Duration
1	Peres J. et al., 2017 (34)	Portugal	Cost-utility	Hospital	6 patients (5 females, 1 male)	N.R.	Rituximab Treatment	Acetylcolinesterase Inhibitors, Plasma exchange (PLEX), Intravenous immunoglobulin (IVIg), Prednisone, Azathioprine, Mycophenolate Mofetil, Cyclosporine	February 2010 - September 2015
2	Heatwole C. et al., 2011 (11)	USA	Cost-minimization	Hospital	Patients with Myasthenia Gravis (MG)	N.R	IVIg	PLEX	Duration: 5 days
3	Bugge C. et al., 2025(30)	Norway	Cost-analysis	Healthcare System	1083 patients,155 with IVIg treatment	N.R	IVIg treatment	Non IVIg treatments	1 January 2010 - 31 December 2021

4	Philips G. et al., 2022(12)	USA	Cost-analysis	Healthcare System	41,940 patients	N.R	Direct cost of gMG standard-of-care therapies and add-on therapies	Full cost of treatment between newly diagnosed, previously diagnosed (PD), exacerbation and crisis event subgroups	1 January 2014 - 31 December 2019
5	Lehnerer S. et al, 2025(28)	Germany	Cost-description	Healthcare System	1660 and 750 MG patients	N.R	Total cost of hospitalization.	No MG-related treatment; Standard treatment; Intensified treatment	From 2014 to 2019
6	Ignatova V. et al., 2022(35)	Bulgaria	Cost-of-illness	Healthcare System	54 adult patients	N.R	Direct annual costs of MG	Indirect costs of MG	May 2020 - September 2020
7	Piehl F. et al, 2024 (32)	Denmark, Finland, Sweden	Cost-description	Healthcare System	8.622 patients with MG; Patients ≥ 18 years old with ≥ 2 MG diagnosis codes.	3%	Total hospitalization costs, healthcare resource utilization (HCRU), direct medical costs	Indirect societal costs	From 2000 to 2020

8	Donin G. et al., 2024 (36)	Czech Republic	Cost-description	Healthcare System	Adult patients with MG	N.R	Outpatient Medication Costs	Outpatient IVIg and PLEX Treatment Costs	January 2017 - December 2020
9	Chen J. et al., 2020 (24)	China	Cost-description	Healthcare System	59,243 MG patients	N.R	Total cost of hospitalization	N.R	1 January 2016 - 31 December 2018
10	Castillo R. et al., 2022(13)	USA	Cost-description	Healthcare System	148 MG patients who received thymectomy.	N.R	Minimally invasive thymectomy (MIS) using video-assisted thoracoscopic surgery (VATS).	Open thymectomy (OT), via sternotomy.	From 2013 to 2018
11	Cai Q. et al., 2024 (33)	Sweden	Cost-analysis	Healthcare System	1275 patients	N.R	All-cause costs, MG-related costs, direct medical costs, indirect costs	Healthcare costs between newly diagnosed patients and patients with preexisting MG	1 January 2010 - 31 December 2017

12	Pisc J. et al., 2023 (14)	USA	Cost-analysis	Healthcare System	3218 patients with MG	N.R	All-cause costs, MG-related costs inpatient and outpatient costs	Healthcare costs between patients with different insurance types (commercial, Medicare, Medicaid)	1 January 2008 - 30 September 2019
13	Souayah N. et al., 2009 (15)	USA	Cost-description	Hospital	651 patients from 1991-1992; 994 patients from 2001-2002	N.R	Total cost of hospitalization	N.R	1991 - 2002
14	Antonini G. et al, 2023 (37)	Italy	Cost-description	Healthcare system	4397 patients	N.R	MG treatments	Non-MG population	January 2011 - December 2018
15	Ting A. etal, 2023 (16)	USA	Cost-description	Healthcare system	1498 adults (aged ≥18 years)	N.R	The second-line therapies for MG	N.R	2 years post-initiation of second-line therapy
16	Furlan C. J. et al, 2016 (38)	Canada	Cost-minimization	Hospital	70 patients with MG (32 IVIg; 38 PLEX)	N.R	PLEX	IVIg	2007 - 2010

17	Schepelmann K. etal, 2010(29)	Germany	Cost-of-illness	Healthcare system	107 patients diagnosed with ALS, FSHD or MG (18 years of age or older)	N.R	Total annual healthcare costs for MG	N.R	12-month period
18	Sonkar KK. etal, 2016 (39)	India	Cost-analysis	Tertiary care center	66 patients with MG (39 males)	N.R	The direct cost of treatment	Indirect cost of MG	1 year (2014-2015)
19	Shen Sh.P. etal, 2023 (25)	Taiwan	Cost-of-illness	Healthcare system	2537 patients with gMG	N.R	HRU and costs in gMG patients	HRU and costs in individuals without gMG	01 January 2019 - 31 December 2019
20	Shen Sh.P. etal, 2023 (25)	USA	Cost-analysis	Medical/pharmacy	1,288 patients with MG	N.R	Full costs of treatments	N.R	June 1, 2008-June 30, 2010

21	Fang W. etal, 2020 (26)	China	Cost-analysis	Hospital/ Healthcare system	4130 patients with JBI (job based insurance) and 472 with RBI (residence based insurance)	N.R	Medical costs of MG patients with JBI	Medical costs of MG patients with RBI	17-year period, from 2000 to 2017
22	Zhdanava M. etal, 2024 (18)	USA	Cost-analysis	Healthcare system	2739 patients and 1638 patients diagnosed with generalized myasthenia gravis (gMG)	N.R	Patients with high follow-up costs	Patients with not-high follow-up costs	1 January 2018-31 December 2021
23	Van Enkhuizen J. et al, 2024 (40)	England	Cost-of-illness	Healthcare system	9087 adult patients with MG	N.R	Treatments used for MG and associated healthcare costs in MG patients	Treatments used for MG and associated healthcare costs in MG patients	Follow up from diagnosis to June 2021 or death
24	Yu J. et at., 2025 (27)	China	Cost-of-illness	Societal	1020 patients with MG	N.R	Annual direct costs of MG	Annual indirect costs of MG	12 months per patient

25	Guptill J.T. et al ., 2012 (19)	USA	Cost-analysis	Healthcare system	113 MG patients and 339 non-MG patients	N.R	IVIg cost	PLEX, pharmacological therapies costs	2009-2011
26	Omorodion J.O. et al, 2017 (20)	USA	Cost-analysis	Healthcare system	Patients hospitalized for MG	N.R	Standard inpatient management, hospital-based care for MG, diagnostic and treatments such as IVIG or plasma exchange	Multiple Sclerosis hospitalizations and all U.S. hospital admissions	2003-2013
27	Zhdanava M. et al ., 2024 (21)	USA	Cost-analysis	Healthcare system	2,739 patients	N.R	IVIg	Other therapies (Non-steroidal immunosuppressives , Cholinesterase inhibitors, Corticosteroids)	1 January 2017 -31 December 2021

Abbreviations: gMG-generalized myasthenia gravis; HCRU-healthcare resource utilization; IVIg- Intravenous immunoglobulin; JBI-job based insurance; MIS-Minimally Invasive Surgery; MG- Myasthenia Gravis; N.R-Not reported; OT-Open thymectomy; PLEX-Plasma exchange; RBI-residence based insurance; VATS- video-assisted thoracoscopic surgery.
