# Supplementary material for: Health economic evaluations of myasthenia gravis: a systematic review
Source: Croat Med J. 2025 Dec;66(6):436–45. doi: 10.3325/cmj.2025.66.436 (PMC12836010; doi:10.3325/cmj.2025.66.436)
Supplement: Supplementary Table 2 [file CroatMedJ_66_s004.pdf]

Supplemental Table 2. Economic data from the selected health economic studies (excluding cost-effectiveness analyses)

No.	Reference	Country/ Currency	Type of pharmacoeconomic study	ICER/ QALY/ QALD	Outcomes	Current values in original currency	Current values in current (EUR) currency
1	Peres J. et al, 2017 (34)	Portugal	Cost-utility	Increase in QALY gained +1.76 per patient	Pre-treatment costs (year before RTX): €121,265/patient<br><br>Post-treatment costs (year after RTX): €107,806/patient<br><br>Cost reduction after RTX treatment: €2243/patient in the first year	Total cost before RTX treatment (year before): €149,982/patient<br><br>Total cost after RTX treatment (first year): €133,296/patient<br><br>Cost reduction in the first year: €2774/patient	Total cost before RTX treatment: €149,982/ patient (year before)<br><br>Total cost after RTX treatment: €133,296/patient (first year)<br><br>Cost reduction: €2774/patient in the first year
2	Heatwole C. et al, 2011 (11)	USA	Cost-minimization	N.R.	Average short-term cost/patient:<br>PLEX: $101,140<br>IVIg: $78,814<br><br>IVIg is cost-minimizing, saving $22,326/patient compared with PLEX	Average short-term cost/patient:<br>PLEX: $140,767<br>IVIg: $109,734<br><br>Cost difference: IVIg saves	Average short-term cost/patient:<br>PLEX: ~€121,400<br>IVIg: ~€94,600<br><br>Cost difference: IVIg saves ~€26,800/patient

						$31,033/patient	
3	Bugge C. et al, 2025(30)	Norway	Cost-analysis	N.R.	Year 1 after MG diagnosis:<br>IVIG patients: €35,714<br>Non-IVIG patients: €15,457<br>Cost ratio: 2.3× higher for IVIG patients<br><br>Year 2:<br>IVIG patients: €19,119<br>Non-IVIG patients: €6256<br>Cost ratio: 3.1× higher for IVIG patients<br><br>Year 5:<br>IVIG patients: €9953<br>Non-IVIG patients: €5634	Year 1 post-diagnosis:<br>IVIG patients: €35,714<br>Non-IVIG patients: €15,457<br><br>Year 2 post-diagnosis:<br>IVIG patients: €19,119<br>Non-IVIG patients: €6256<br><br>Year 5 post-diagnosis:<br>IVIG patients: €9953<br>Non-IVIG patients: €5634	Year 1 post-diagnosis:<br>IVIg patients: €35,714<br>Non-IVIg patients: €15,457<br><br>Year 2 post-diagnosis:<br>IVIg patients: €19,119<br>Non-IVIg patients: €6256<br><br>Direct medical costs: 3.1× higher for IVIg patients<br><br>Year 5 post-diagnosis:<br>IVIg patients: €9953<br>Non-IVIg patients: €5634

4	Philips G. et al, 2022(12)	USA	Cost-analysis	N.R.	Annual costs per patient: ND $26,419; PD $24,941; Exacerbation $43,734<br><br>Crisis costs: Pre-crisis $49,236; Post-crisis $173,956<br><br>Direct medical costs: ND $9890; PD $9186; Exacerbation $21,550<br><br>Drug cost contribution: ND 52.8% ($5224); PD 73.4% ($6743)	Mean all-cause costs per patient: ND $29,031; PD $27,388; Exacerbation $48,058<br><br>Mean direct costs: ND $10,865; PD $10,091; Exacerbation $23,669<br><br>Drug costs (IVIg and SCIg): ND 52.8% ($5735); PD 73.4% ($7407)	Mean all-cause annual costs per patient (2024 EUR): ND ~€24,570; PD ~€23,196; Exacerbation ~€40,670<br><br>Mean direct medical costs: ND ~€9200; PD ~€8540; Exacerbation ~€20,040<br><br>Drug costs (IVIg and SCIg) share of direct costs: ND 52.8% (~€4,860); PD 73.4% (~€6,270)
5	Lehnerer S. et al, 2025(28)	Germany	Cost-description	N.R.	Direct costs per patient/year:<br>-Inpatient care: €6634 (95% CI: €6628–€6640)<br>-Outpatient medication: €658 (95% CI: €656–€660)	Inpatient care: €6634 (95% CI: €6628–€6640)<br><br>Outpatient medication: €658 (95% CI: €656–€660)	The same as the previous column

					Medical aids/remedies: €1128 (95% CI: €1126–€1131)	Medical aids/remedies: €1128 (95% CI: €1126–€1131)	
6	Ignatova V. et al, 2022(35)	Bulgaria	Cost-of-illness	N.R.	Median annual cost per patient: €4047<br><br>Direct cost components:<br>Hospitalization: €1512<br>Outpatient visits: €194<br><br>Cost comparison by thymectomy status:<br>With thymectomy: €3047<br>Without thymectomy: €825<br><br>Indirect costs: up to €5665	Median annual cost per patient: €4454<br><br>Direct costs breakdown:<br>Drugs = largest cost component<br>Hospitalization: €1665<br>Visits: €213<br><br>Cost comparison by thymectomy status:<br>With thymectomy: €3354	The same as the previous column

						Without thymectomy: €908<br><br>Indirect costs (total): €6239	
7	Piehl F. et al, 2024 (32)	Denmark, Finland, Sweden	Cost-description	N.R.	Total annual cost/ patient:<br>Denmark: €12,185<br>Finland: €9036<br>Sweden: €5997<br><br>Direct costs (share of total):<br>Denmark: €6721 (55%)<br>Finland: €6360 (70%)<br>Sweden: €3318 (55%)	Direct annual cost/patient:<br>Denmark: €6930 (55%)<br>Finland: €6557 (70%)<br>Sweden: €3421 (55%)<br><br>Total annual cost/patient:<br>Denmark: €12,563<br>Finland: €9316<br>Sweden: €6183	The same as the previous column

						Very severe MG annual cost: €20,177–€34,531 per patient/year across Denmark, Finland, and Sweden	
8	Donin G. et al, 2024 (36)	Czech Republic	Cost-description	N.R.	Most common & least expensive treatments:<br>AChE inhibitors: €305/year per patient<br>Corticosteroids: €50/year per patient<br><br>Higher-cost therapies:<br>Immunosuppressants: €842/year per patient<br>Biologicals (rare use): €33,336/year per patient<br><br>Intensive, high-cost therapies (used less frequently):<br>IVIg: €20,700/year per patient<br>PLEX: €18,206/year per patient	Most common & lowest-cost MG treatments:<br>AChE-I: ~€314/year per patient<br>Corticosteroids: ~€52/year per patient<br><br>More costly therapies:<br>Immunosuppressants: ~€868/year per patient<br>Biological therapies (rare use):	The same as the previous column

						~€34,368/year per patient<br><br>Intensive, high-cost treatments: IVIg: ~€21,342/year PLEX: ~€18,771/year	
9	Chen J. et al, 2020 (24)	China	Cost-description	N.R.	Median hospitalization cost per MG patient: $1037 (IQR $493–$2925)<br><br>Adults incur higher hospitalization costs than juveniles: $1433 vs. $786	Median hospitalization cost: $1250 per admission (IQR $594–$3526)<br><br>Adults incurred higher costs than juveniles: $1728 vs. $947	Median hospitalization cost: €913 (IQR €434–€2574)<br><br>Higher costs for adults vs. juveniles: €1261 vs. €692
10	Castillo R. et al, 2022(13)	USA	Cost-description	N.R.	Median Total Cost (adjusted to 2019 USD):MIS: $18,431; OT: $22,121;	Total cost (adjusted to 2025 USD):MIS: median $20,247; OT: median $24,289;	Median Total Cost (adjusted to 2025 EUR):MIS: €19,200; OT: €23,000

11	Cai Q. et al, 2024 (33)	Sweden	Cost-analysis	N.R.	Year 1 cost gap ND vs PE MG: +€7302, mainly inpatient (€6275)<br><br>Year 1 MG-related gap: +€6188 (inpatient €5198)<br><br>Age impact (Year 1):<br>65 yrs: +€6337<br>18–65 yrs: +€8427<br><br>Total Year 1 costs: ND €19,900 vs PE €9951<br><br>Year 2 ND costs: €11,422 total	Year 1 all-cause cost difference (ND vs PE MG): €7521, mainly inpatient (€6464)<br><br>Year 1 MG-related cost difference: €6382, inpatient €5355<br><br>Age-based Year 1 differences:<br>65 yrs: €6534<br>18–65 yrs: €8684<br><br>Total Year 1 costs:<br>ND MG: €20,498 (Indirect: €7724)<br>PE MG: €10,250 (Indirect: €4108)	The same as the previous column

						Year 2 ND costs: €11,771 total, indirect €5933	

12	Pisc J. et al, 2023 (14)	USA	Cost-analysis	N.R.	All-cause costs: Commercial $41,194 vs Medicare $34,802<br><br>MG-related costs: Commercial $39,079 vs Medicare $33,446<br><br>ED/ICU costs: ED $8143 vs $5179; ICU $2174 vs $1624<br><br>Outpatient: Commercial $10,752 vs $5304<br><br>Inpatient: Commercial $19,257 vs $22,009	All-cause: $43,334 vs $36,602<br>MG-related: $41,126 vs $35,175<br>ED/ICU: $8567/$2286 vs $5448/$1708<br>Outpatient: $11,306 vs $5577<br>Inpatient: $20,254 vs $23,150	All-cause costs: €39,500 vs €33,400<br>MG-related costs: €37,500 vs €32,100<br>ED/ICU costs: €7800/€2100 vs €4900/€1540<br>Outpatient costs: €10,300 vs €5000<br>Inpatient costs: €18,400 vs €20,700
13	Souayah N. et al, 2009 (15)	USA	Cost-description	N.R.	Hospitalization charges/patient: $84,100 (1991–1992) → $118,000 (2001–2002, +51%)<br><br>Average daily charges: $3932 → $5500<br><br>Urban teaching hospitals: Stable costs (~$74,500 → ~$75,000)	Hospitalization charges/patient: $163,000 → $202,960 (1991–1992 to 2001–2002, 2025 USD)<br><br>Average daily charges: $7620 → $9460	Hospitalization charges/patient: €89,200 → €125,000 (1991–1992 to 2001–2002, +51%)<br><br>Average daily charges: €4170 → €5840<br><br>Urban teaching hospitals: Stable €79,200 → €79,700

					Non-teaching hospitals: Increased from $58,000 → $118,300	Urban teaching hospitals: Stable ~$128,700 → ~$129,000<br><br>Non-teaching hospitals: Increased ~$112,000 → $203,476	Non-teaching hospitals: Increased €61,700 → €125,500
14	Antonini G. et al, 2023 (37)	Italy	Cost-description	N.R.	Mean annual cost: €3771 (hospitalization & drugs), decreases over time<br><br>By treatment line: €2007 → €7019 (fourth line)<br><br>Exacerbations: €7827 first year, then lower<br><br>Non-MG patients: €869/year	Mean annual cost (MG patients): ~€3978 (mainly hospitalization & drugs), decreasing over time<br><br>By treatment line: €2117 (first) → €7403 (fourth, driven by drugs & outpatient)<br><br>Exacerbations: €8258 first	The same as previous column

						year, declining thereafter<br><br>Non-MG patients: ~€916/year	

15	Ting A. etal, 2023 (16)	USA	Cost-description	N.R.	Mean MG-related cost: $69,206 (Inpatient $20,495, Outpatient $38,828, Pharmacy $9882)<br><br>Median cost: $12,201 (Inpatient $0, Outpatient $2818, Pharmacy $1196)<br><br>Year 1 total population: $38,247 / $4,140 (mean/median)<br><br>Chronic IVIg (2 yrs): $243,849 / $177,480; Year 1: $134,693 / $111,651; Year 2: $109,157 / $63,386	Mean total cost: $72,741 (Inpatient $21,555, Outpatient $40,814, Pharmacy $10,388)<br><br>Median total cost: $12,837 (Inpatient $0, Outpatient $2963, Pharmacy $1258)<br><br>Year 1 / Year 2 (mean/median) : $40,223 / $4353 ; $32,547 / $2540<br><br>Chronic IVIg 2-yr: $256,383 / $186,505 (Year 1: $141,689 / $117,413; Year	Mean MG-related cost/patient: €65,300 (Inpatient €19,350, Outpatient €36,650, Pharmacy €9320)<br><br>Median cost: €11,520 (Inpatient €0, Outpatient €2660, Pharmacy €1130)<br><br>Yearly costs (total population): Year 1 €36,100 / €3910; Year 2 €29,200 / €2280<br><br>Chronic IVIg subgroup (2 yrs): €233,000 / €169,700 Year 1: €128,700 / €106,700<br>Year 2: €104,300 / €60,500

						2: $114,694 / $66,653)	
16	Furlan C. J. et al, 2016 (38)	Canada	Cost-minimization	N.R.	Overall costs: IVIg $8309 (mainly blood products $6823); PLEX $6271 (mainly hospital $4628)<br><br>Cost-effectiveness: PLEX is short-term cost-minimizing for insured patients	IVIg total cost: ~$10,428 (blood products $8563)<br><br>PLEX total cost: ~$7868 (hospital $5808)	IVIg total cost: €7150 (blood products €5870)<br><br>PLEX total cost: €5400 (hospital €3990)

17	Schepelmann K. etal, 2010(29)	Germany	Cost-of-illness	N.R.	Total annual MG cost: €14,950<br><br>Direct costs: €11,840; Indirect costs: €2790<br><br>Drugs: ~€1800/year per patient	Total annual MG cost: ~€19,435<br><br>Direct costs: ~€15,392; Indirect costs: ~€3627<br><br>Drugs: ~€2340/year per patient	The same as the previous column
18	Sonkar KK. etal, 2016 (39)	India	Cost-analysis	N.R.	Total annual MG cost: median INR 61,390 (~US$912)<br><br>Outpatient: ~INR 20,440 (~US$304) | Inpatient: ~INR 44,312 (~US$658) | ICU: ~INR 59,574 (~US$885)<br><br>Thymectomy: ~INR 45,000 (~US$668) | IVIg/PLEX: median ~INR 378,930 (~US$5627)<br><br>Indirect costs: median ~INR 5100 (~US$76)	Total annual MG cost: median INR 104,364 (~$1144)<br><br>Outpatient/Inpatient/ICU: ~INR 34,748 / 75,330 / 101,276 (~$381 / $826 / $1110)<br><br>Thymectomy: ~INR 76,500 (~$838) | IVIg/PLEX: median ~INR	Total annual MG cost: median €685 (range €51–€5950)<br><br>Outpatient: median €228 | Inpatient: median €333 | ICU: median €448<br><br>Thymectomy: ~€340 | IVIg/PLEX: median €4235<br><br>Indirect costs: median €38 | Stroke inpatient: median €157

						644,181 (~$7058)<br><br>Indirect costs: ~INR 8670 (~$95)	
19	Shen Sh.P. etal, 2023 (25)	Taiwan	Cost-of-illness	N.R.	Mean total healthcare cost (gMG patients): NT$ 135,219 (~US$ 4456)<br><br>Mean total healthcare cost (comparator group): NT$ 40,904 (~US$ 1348)<br><br>Mean difference (all-cause costs): NT$ 94,997 (~US$ 3133 ± 2521)	Mean total healthcare cost (gMG patients): NT$140,628 (~$4678)<br><br>Mean total healthcare cost (comparators): NT$42,541 (~$1415)<br><br>Mean difference (all-cause costs): NT$98,796 (~$3290 ± $2647)	Mean total healthcare cost (gMG patients): ≈ €4035<br><br>Mean total healthcare cost (comparators): ≈ €1218<br><br>Mean difference (all-cause costs): ≈ €2835 (± €2282)

20	Shen Sh.P. etal, 2023 (25)	USA	Cost-analysis	N.R.	Average annual cost per MG patient: $24,988 (median $9023)<br><br>Hospital costs: 27% of total<br>Highest costs: Age 20–39<br><br>Pharmacy costs: $9.4M/year, IVIg 85% (~$8M)<br><br>Mean cost per treatment: IVIg $4663; PLEX $949	Avg. annual cost per MG patient: ~$32,985 (median $11,909)<br><br>Hospital vs. non-hospital: 27% vs. 73%<br>Highest costs: ages 20–39<br><br>Pharmacy costs: ~$12.4M/year, IVIg 85% (~$10.56M)<br><br>Treatment cost: IVIg ~$6156/infusion; PLEX ~$1252/session	Average annual cost per MG patient: ≈ €28,367 (median ≈ €10,244)<br><br>Hospital vs. non-hospital spending: 27% vs. 73%<br>Highest-cost age group: 20–39 years<br><br>Annual pharmacy expenditure: ≈ €10.66M, with IVIg representing ~85% (~€8.91M)<br><br>Treatment costs: IVIg ≈ €5296 per infusion; PLEX ≈ €1077 per session

21	Fang W. etal, 2020 (26)	China	Cost-analysis	N.R.	MG patients with Juvenile Brain Injury(JBI):<br>Outpatient: $171<br>Clinic visit: $35<br>Inpatient: $3539<br>Hospitalization: $2526<br><br>MG patients with Recurrent Brain Injury(RBI):<br>Outpatient: $209<br>Clinic visit: $42<br>Inpatient: $4224	MG patients with JBI:<br>Outpatient visit: $195<br>Clinic visit: $40<br>Inpatient stay: $4034<br>Hospitalization: $2879<br><br>MG patients with RBI:<br>Outpatient visit: $239<br>Clinic service: $48<br>Inpatient stay: $4814<br>Hospitalization: $3045	Costs for MG patients with JBI<br>Outpatient visit: ≈ €168<br>Clinic visit: ≈ €34<br>Inpatient stay: ≈ €3469<br>Hospitalization: ≈ €2475<br><br>Costs for MG patients with RBI<br>Outpatient visit: ≈ €206<br>Clinic service: ≈ €41<br>Inpatient stay: ≈ €4140<br>Hospitalization: ≈ €2619
22	Zhdanava M. etal, 2024 (18)	USA	Cost-analysis	N.R	High-cost gMG patients:<br>Median annual healthcare cost: > $110,000	High-cost gMG patients:<br>Median annual healthcare cost: > $115,500<br><br>High-cost criteria (monthly cost):	High-cost gMG patients:<br>Median annual healthcare cost: €102,300<br><br>High-cost criteria (monthly cost):<br>Main cohort: ≥ €8745<br>Subgroup: ≥ €8519

						Main cohort: ≥ $9874<br>Subgroup: ≥ $9617	
23	Van Enkhuizen J. et al, 2024 (40)	England	Cost-of-illness	N.R	Mean costs per patient-year: Inpatient £3226, Outpatient £454, A&E £140<br><br>Therapies: IVIg £1514, PLEX £4233, Rituximab £1811<br><br>Total secondary care: £3820 (~$4849)	Mean costs per patient-year: Inpatient £3355, Outpatient £472, A&E £146<br><br>Therapies: IVIg £1575 PLEX £4402, Rituximab £1883<br><br>Total secondary care cost: £3973 (~$5091)	Mean costs per patient-year: Inpatient ≈ €3805, Outpatient ≈ €536, A&E ≈ €165<br><br>Therapies: IVIg ≈ €1786, PLEX ≈ €4994, Rituximab ≈ €2137<br><br>Total secondary care cost: ≈ €4510 per patient-year

24	Yu J. et al, 2025 (27)	China	Cost-of-illness	N.R.	Median annual costs per patient:<br><br>Direct medical: $1860.2<br>Medication: $1167.9<br><br>Direct non-medical: $248.2	Median annual costs per patient:<br><br>Direct medical: $1860.2<br>Medication: $1167.9<br><br>Direct non-medical: $248.2	Median annual costs per patient:<br><br>Direct medical: ≈ €1730 (USD 1860.2)<br>Medication: ≈ €1086 (USD 1167.9)<br><br>Direct non-medical: ≈ €231 (USD 248.2)
25	Guptill J.T. et al, 2012 (19)	USA	Cost-analysis	N.R.	Mean annual cost: MG $20,190 vs. controls $4515<br><br>Pharmacy: MG $9012 vs. $608<br><br>Non-pharmacy: MG $11,178 vs. $3958<br><br>MG-attributable cost: $15,675	Mean annual costs: MG ~$27,863 vs. controls ~$6231<br><br>MG-attributable costs: $21,621<br><br>Pharmacy costs: MG $12,436 vs. $839<br><br>Non-pharmacy costs: MG $15,423 vs. $5460	Mean annual cost: MG ≈ €25,335; Controls ≈ €5660<br><br>MG-attributable cost: ≈ €19,671<br><br>Pharmacy costs: MG ≈ €11,312; Controls ≈ €763<br><br>Non-pharmacy costs: MG ≈ €14,028; Controls ≈ €4961

26	Omorodion J.O. et al, 2017 (20)	USA	Cost-analysis	N.R.	Total inpatient charges for MG: Increased 13-fold, from ~$41.8M → ~$546.8M<br><br>MG-related hospital discharges: Increased over 6-fold, from 870 → 5535	Total inpatient charges for MG: 13-fold increase, from ~$51.9M → $678.0M<br><br>MG discharges: Increased over 6-fold, from 870 → 5535	Total inpatient charges for MG: From ~€60.8M → ~€794.3M<br><br>MG discharges: Increased over 6-fold, from 870 → 5535
27	Zhdanava M. et al, 2024 (21)	USA	Cost-analysis	N.R.	MG treatment costs highest in ages 20–39 ($37,522 total).<br><br>Total MG pharmacy costs: $9.4M; IVIg = 85% (~$8M).	MG treatment costs by age: 0–19: ~$8143; 20–39: ~$38,647; 40–64: ~$28,439; 65+: ~$21,307<br><br>Total annual MG pharmacy costs: ~$9.7M	MG treatment costs by age (€): 0–19: 7353; 20–39: 34,905; 40–64: 25,682; 65+: 19,238<br><br>Total pharmacy costs: ~€8.74M (IVIg ~€7M)

Abbreviations: ICER- Incremental Cost-Effectiveness Ratio; N.R- Not reported; QALD- Quality-Adjusted Life Day ;QALY- Quality-Adjusted Life Year.
